# RGGC-UNet: Accurate Deep Learning Framework for Signet Ring Cell Semantic Segmentation in Pathological Images

**DOI:** 10.3390/bioengineering11010016

**Published:** 2023-12-23

**Authors:** Tengfei Zhao, Chong Fu, Wei Song, Chiu-Wing Sham

**Affiliations:** 1 School of Computer Science and Engineering, Northeastern University, Shenyang 110819, China; 2 Engineering Research Center of Security Technology of Complex Network System, Ministry of Education, Shenyang 110819, China; 3 Key Laboratory of Intelligent Computing in Medical Image, Ministry of Education, Shenyang 110819, China; 4 School of Computer Science, The University of Auckland, Auckland 1142, New Zealand

**Keywords:** semantic segmentation, signet ring cell, residual ghost block, ghost coordinate attention

## Abstract

Semantic segmentation of Signet Ring Cells (SRC) plays a pivotal role in the diagnosis of SRC carcinoma based on pathological images. Deep learning-based methods have demonstrated significant promise in computer-aided diagnosis over the past decade. However, many existing approaches rely heavily on stacking layers, leading to repetitive computational tasks and unnecessarily large neural networks. Moreover, the lack of available ground truth data for SRCs hampers the advancement of segmentation techniques for these cells. In response, this paper introduces an efficient and accurate deep learning framework (RGGC-UNet), which is a UNet framework including our proposed residual ghost block with ghost coordinate attention, featuring an encoder-decoder structure tailored for the semantic segmentation of SRCs. We designed a novel encoder using the residual ghost block with proposed ghost coordinate attention. Benefiting from the utilization of ghost block and ghost coordinate attention in the encoder, the computational overhead of our model is effectively minimized. For practical application in pathological diagnosis, we have enriched the DigestPath 2019 dataset with fully annotated mask labels of SRCs. Experimental outcomes underscore that our proposed model significantly surpasses other leading-edge models in segmentation accuracy while ensuring computational efficiency.

## 1. Introduction

Signet ring cell carcinoma (SRCC) represents a relatively uncommon subtype of profoundly aggressive adenocarcinoma [1]. Predominantly encountered within the gastric glandular cells, primary SRCCs exhibit a notable association with gastric malignancies [2]. In the SRCC, a signet ring cell (SRC) contains a lot of mucins that push the nucleus to the periphery [3]. Moreover, SRCC has the highest malignancy and poorest prognosis in advanced gastric cancer. The prompt and precise diagnosis followed by timely intervention of SRCs in the gastric region can substantially enhance patients’ survival rates. In the realm of the digestive system, the gold standard for diagnosing SRCC is the examination of pathological images [4]. Therefore, detecting the SRCs in pathological images is essential for diagnosing SRCC. Nevertheless, the conventional manual segmentation of signet ring cells is susceptible to time-consuming processes and human error. Automatic segmentation methods have, therefore, been devised to enhance both accuracy and efficiency. These methods typically involve using image processing and machine learning algorithms to identify and segment signet ring cells from surrounding tissue or other types of cells. By automating the segmentation process, medical professionals can quickly and accurately analyze large amounts of data, leading to earlier detection and improved treatment of cancer. Hence, the computer-aided diagnosis-based analysis of SRC, serving as a supplementary investigation, holds significant promise and is in high demand.

The Digestive-System Pathological Detection and Segmentation Challenge of 2019, abbreviated as DigestPath 2019, marks the inaugural competition and open dataset dedicated to the detection of signet ring cells (SRCs) within pathological images [5]. Automatic SRC detection algorithms had not been thoroughly investigated prior to this challenge. As a result, the DigestPath 2019 challenge has driven research into the SRC detection algorithms. Unfortunately, only a portion of the data has been annotated, and the algorithms for this research are all based on semi-supervised object detection methods [4,6,7,8,9,10]. Therefore, existing semi-supervised detection labels in the DigestPath 2019 dataset have not led to an increase in the network performance, limiting the application in practical medicine.

In recent years, deep learning methods [11,12,13,14,15,16,17,18] have achieved success in medical image analysis, such as biomedical segmentation and nuclei instance segmentation [19,20,21,22,23,24]. Most of this research is based on convolutional neural networks (CNNs) and has performed well in diverse biomedical segmentation applications. As an illustration, Lu et al. [20] presented an enhanced algorithm that employs a collaborative optimization approach involving multiple-level set functions. This method is designed for the segmentation of cytoplasm and nuclei in cases where cervical cells overlap and form clumps. Chen et al. [21] introduced the concept of deep contour-aware networks for precise gland segmentation, abbreviated as DCAN. This framework generates precise probability maps for glands while simultaneously delineating accurate contours, enabling effective separation of clustered objects and thereby enhancing gland segmentation performance. Naylor et al. [22] introduced an innovative approach involving fully convolutional networks designed for the automated segmentation of nuclei within histopathology data stained with hematoxylin and eosin (H&E). Their methods address the challenge of segmenting touching nuclei by treating the problem as a regression task for distance maps, thereby providing a solution to this segmentation issue. Simon et al. [23] introduced the HoVer-Net, a novel approach designed for both simultaneous nuclei segmentation and classification. This method harnesses the wealth of information embedded in the vertical and horizontal distances from nuclear pixels to their respective centers of mass. Zhou et al. [25] proposed the CIA-Net, which incorporates a multi-level information aggregation module between two task-specific decoders. This approach exploits the advantages of spatial and texture dependencies between nuclei and contours by bidirectionally aggregating task-specific features. Unfortunately, these methods suffer from model redundancy, resulting in low efficiency.

Hence, lightweight deep learning frameworks have become another topic of study. In particular, lightweight deep learning frameworks have been applied to medical image analysis. For instance, Zhang et al. [26] proposed a lightweight hybrid convolutional network for liver tumor segmentation. Zhao et al. [27] introduced a streamlined feature attention network to segment both nucleus and cytoplasm regions within cervical images. Unfortunately, the above methods suffer from insufficient model expression ability, resulting in low accuracy. In addition, these methods cannot be used directly in actual medical scenarios because they are low in efficiency and accuracy.

The segmentation of SRCs poses a challenge that remains unaddressed in current research, primarily because of the absence of reliable ground truth for SRCs. This deficiency has notably hampered advancements in the field of SRC segmentation. In the clinical diagnosis, pathologists rely on the presence of a substantial number of SRCs within pathological Whole Slide Images (WSI) as a key indicator suggesting a higher likelihood of the WSI being of the SRCC type. In this paper, we introduce an efficient and accurate deep learning framework tailored for the semantic segmentation of SRCs in pathology images. In particular, we have fully annotated the mask labels for SRC in the DigestPath 2019 SRC detection dataset [6]. In our approach, we employ an encoder-decoder architecture that incorporates a residual ghost block featuring ghost coordinate attention (GCA). In addition, our proposed encoder enhances the extraction of the features of the SRC boundary region. Our main contributions are summarized as follows.

- We propose an efficient and accurate deep learning framework for signet ring cell semantic segmentation in pathological images.
- We design a novel encoder that not only refines the network’s capability but also notably enhances its performance in segregating overlapping and clustered cells.
- We propose ghost coordinate attention, which can efficiently capture the long-range dependencies.
- We provide full mask labels of SRC on the DigestPath 2019 dataset, referred to as the SRC dataset.
- Our experimental findings validate that the network proposed in this study attains superior evaluation scores and generates more refined segmentation outcomes when compared to other state-of-the-art methods for SRC segmentation.

The structure of this paper is as follows: Section 2 provides an introduction to the proposed method. In Section 3, we present the dataset, evaluation metrics and implementation details related to the experiment. In Section 4, the experimental results are the discussion and analysis. Lastly, Section 5 offers a summary of our work and a brief discussion on potential future research directions.

## 2. Methods

**Figure 1:**
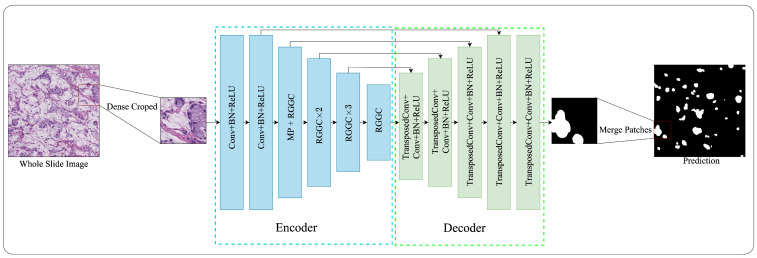
Overview of RGGC-UNet.

Figure 1 provides an overview of our proposed efficient and accurate deep learning framework for SRC semantic segmentation in pathology images. In this study, we begin with 128 × 128 × 3 image patches, which are generated using dense cropped methods to extract relevant features from the original images. Detailed descriptions will be presented in the following subsections.

### 2.1. Network Architecture

**Figure 2:**
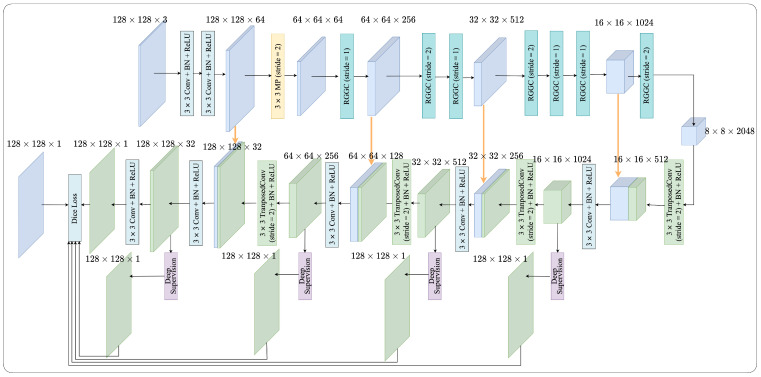
Detailed architecture of RGGC-UNet.

Figure 2 provides a comprehensive depiction of the intricate architecture of the proposed RGGC-UNet. Our proposed network is an adaptation of the UNet framework, comprising an encoder and a decoder designed for the segmentation of SRCs. The encoder is proficient at extracting a highly effective set of features. Meanwhile, the decoder incorporates transposed convolution and 1 × 1 convolution operations.

In the encoder, we incorporate the ghost block with ghost coordinate attention, which is extensively discussed in Section 2.2. Detailed explanations of ghost coordinate attention mechanisms are presented in Section 2.3. Additionally, we delve into the RGGC block in Section 2.4 and the decoder in Section 2.5. The utilization of deep supervision is addressed in Section 2.6. We introduce loss function in Section 2.7.

### 2.2. Encoder

In order to derive a valid set of features from the SRC, we introduce an innovative downsampling mechanism as an integral component of the encoder. The encoder primarily employs a sequence of residual ghost blocks with ghost coordinate attention (RGGC) for the downsampling process.

Our network comprises four downsampling modules, each incorporating a variable number of ghost blocks with ghost coordinate attention (GGC). As illustrated in Figure 2, the initial downsampling module utilizes a 3×3 max pooling (MP) operation followed by an RGGC block. Subsequently, the second and third downsampling modules incorporate two and three stacked RGGC blocks with stride = 2 where an RGGC block performs the downsampling operation, respectively. Meanwhile, the fourth downsampling module solely relies on a RGGC block for the downsampling operation.

Through the utilization of ghost blocks, our network is capable of generating feature-rich maps with significantly fewer input features compared to conventional convolution methods, thus enhancing the computational efficiency of our encoder. Particularly noteworthy is the advantage conferred by ghost coordinate attention (GCA), which empowers our proposed encoder to effectively capture dependence between long-range pixels.

### 2.3. Ghost Coordinate Attention

**Figure 3:**
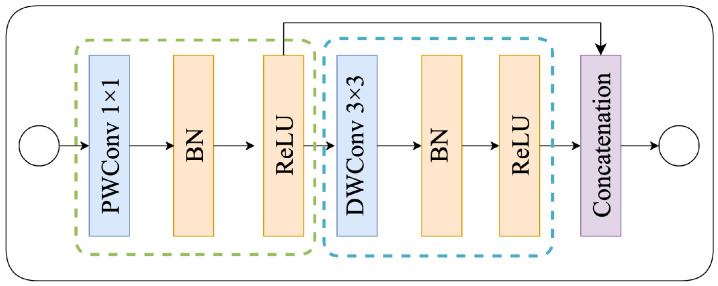
Diagram of ghost block. The green dash box represents identity operation. The blue dash box represents the efficiency operation [28].

Figure 3 depicts the ghost block [28], a novel component in our study. It is well-established that the inclusion of ghost blocks can significantly enhance the feature generation capabilities of a convolutional neural network while maintaining a remarkably lower computational overhead.

This enhancement is achieved through a two-step process within the ghost block. Initially, it generates a set of intrinsic features utilizing a 1×1 point-wise convolution operation. Subsequently, it employs computationally economical operations to further expand the feature set based on these intrinsic features. The resultant feature sets are then concatenated along the channel dimension.

It is worth noting that the computational cost associated with linear operations on feature maps within the ghost block is substantially lower when compared to traditional convolutional techniques, thereby surpassing the efficiency of other existing approaches.

Mathematically, ghost block is defined by

(1) $$Y=Concat(\left[X*F_{1\times 1},\left(X*F_{1\times 1}\right)*F_{dp}\right]),$$

where ∗ denote convolution operation, and $X\in R^{H\times W\times C}$ with height *H*, width *W* and channel’s number *C* is the input feature. $F_{1\times 1}$ and $F_{dp}$ are the 1×1 point-wise and 3×3 depth-wise convolutional filter, respectively. $Y\in R^{H\times W\times C_{out}}$ is the output feature.

Unfortunately, as evident from Equation (1), it becomes apparent that the spatial information is exclusively captured by the cost-effective operations for merely half of the features. The residual features, generated solely through 1×1 point-wise convolutions, lack any form of interaction with neighboring pixels. Consequently, this limited capacity to capture spatial information could potentially hinder the further enhancement of performance.

As aforementioned, the ghost block has previously been identified as having limitations due to its weak ability to capture spatial information, which may negatively impact its performance. However, the proposed ghost coordinate attention (GCA) solves this problem. Our GCA adopts the advantage of coordinate attention [29] and ghost block. While channel attention converts a feature tensor into a single feature vector through 2D global pooling, ghost coordinate attention takes a different approach by breaking down channel attention into two distinct 1D feature encoding processes. These processes aggregate features along two spatial directions separately. As a result of this approach, long-range dependencies are captured effectively along one spatial direction, and, at the same time, precise positional information is carefully preserved along the other spatial directions. The outcome of this process is two separate sets of encoded feature maps, each characterized by its direction awareness and sensitivity to positional information. These feature maps can be applied in a complementary manner to the input feature map, thereby enhancing the representations of the objects of interest.

**Figure 4:**
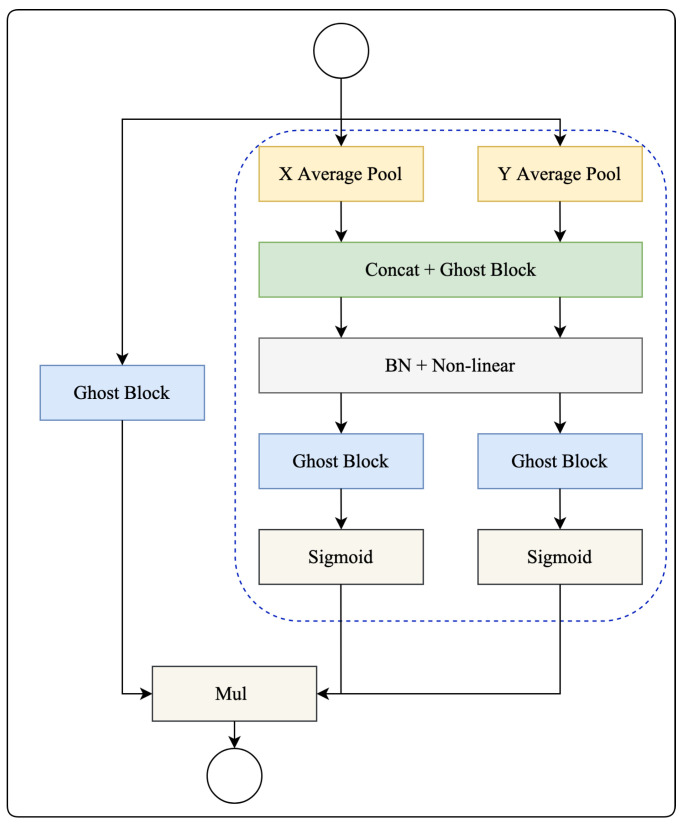
The diagram of GGC block. The blue dash square denotes the ghost coordinate attention (GCA).

In Figure 4, the blue dashed square denotes a comprehensive elucidation of the ghost coordinate attention mechanism. This mechanism adeptly encapsulates both channel interrelations and long-range dependencies, enables a global receptive field, and encodes precise positional information.

Global pooling is a frequently utilized technique in channeling attention to encode spatial information on a broad scale. However, its method of compressing global spatial information into a channel descriptor makes the preservation of positional information challenging. Such positional information is crucial for recognizing spatial structures in vision-related tasks. Attention blocks efficiently capture long-range interactions with accurate positional information. Unlike conventional methods, the X adaptive average pool and Y adaptive average pool aggregate features in two spatial directions. This approach diverges significantly from the squeeze operation seen in channel attention methods, which usually yield a singular feature vector. These transformations facilitate the attention block in encoding long-range dependencies in one spatial direction while maintaining precise positional information in the other. This dual-action allows networks to pinpoint the objects of interest with heightened accuracy.

As explained earlier, the X adaptive average pool and Y adaptive average pool allow for a global receptive field and encapsulate precise positional information. To leverage the high-level representations derived, a method coined as coordinate attention generation is introduced as a subsequent transformation. Specifically, the feature maps amalgamated by the X adaptive average pool and Y adaptive average pool are first concatenated and then subjected to a shared ghost block. The resulting feature map is then divided along the spatial dimension into distinct tensors and dispatched to two separate ghost blocks and sigmoid functions.

In contrast to channel attention, which prioritizes re-calibrating the significance of varied channels, the ghost coordinate attention block also aspires to integrate spatial information. The concurrent application of attention along both horizontal and vertical directions to the input tensor enables each element in the attention maps to signify the presence of an object of interest in the corresponding row and column. Especially, our proposed GCA can enhance the feature generation capability through using ghost blocks. This intricate encoding mechanism empowers the ghost coordinate attention to precisely discern the exact locations of objects of interest, enhancing the model’s overall representation capabilities.

### 2.4. Residual Ghost Block with Ghost Coordinate Attention

**Figure 5:**
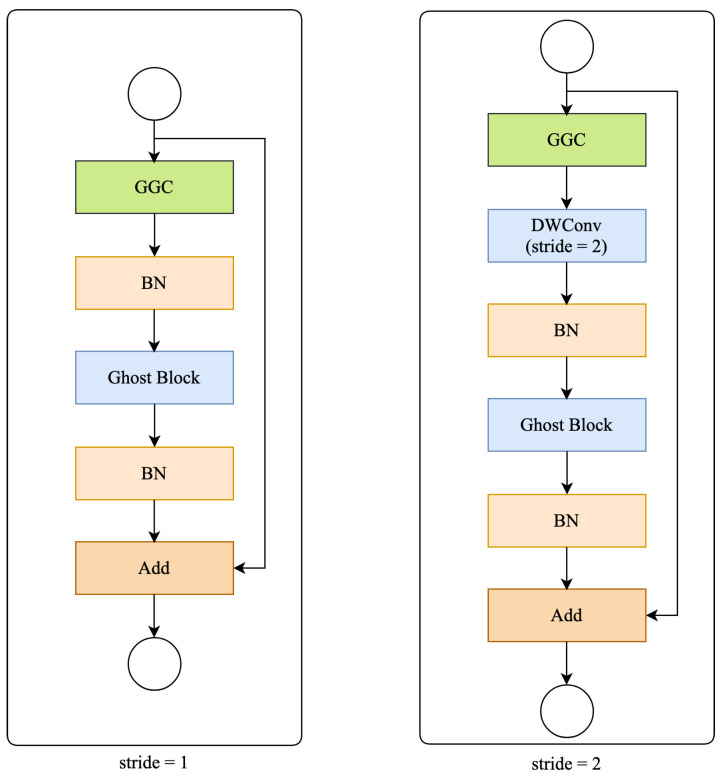
Diagram of an RGGC block.

The residual ghost block with ghost coordinate attention (RGGC), which incorporates the ghost block and GCA is illustrated in Figure 5. A RGGC comprises the residual block consisting of a GGC block and a ghost block. As shown in Figure 4, the GGC block generates expanded features with more channels, while the ghost block reduces the channel count to produce output features. Importantly, the GCA can help a ghost block to preserve information along one spatial direction while precise positional information can be preserved along the other spatial direction.

Figure 4 also shows that the GGC block consists of two parallel branches, a ghost block, and a GCA branch, which extract information from different perspectives. As mentioned earlier, the GCA branch can help the ghost block branch to enhance its representation ability. In the GGC block, the GCA branch operates in parallel with the ghost block branch to enhance the expanded features. Then the output features from GGC block are sent to another ghost block for producing output features. This allows the RGGC block to capture long-range dependence between pixels in different spatial locations and enhance the model’s expressiveness.

### 2.5. Decoder

As depicted in Figure 2, the decoder is constructed with four upsampling modules, employing a combination of transposed convolution and 1 × 1 convolution with Rectified Linear Unit (ReLU) activation. This configuration effectively doubles the spatial resolution of the input data.

The concatenation operation plays a pivotal role in this process by merging the skip and output features of the TransposedConv-ReLU modules. This operation seamlessly integrates the low-level features from the encoder, located at the same level, directly into the decoder at that level. Consequently, it augments the granularity of information within the target region under evaluation. This enhancement in information granularity leads to an improvement in the segmentation performance of the model.

### 2.6. Deep Supervision

**Figure 6:**
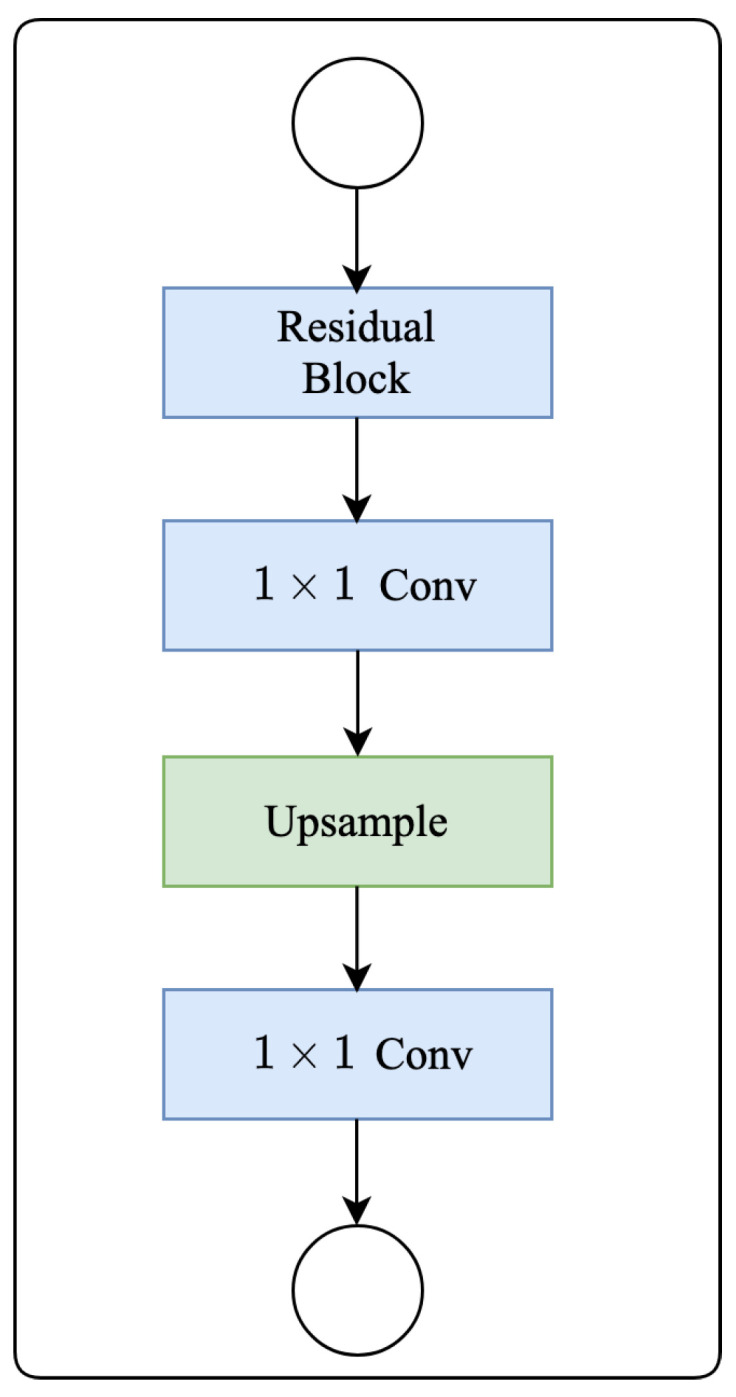
Diagram of deep supervision.

To enhance back-propagation and ensure greater stability in the decoder, we implement deep supervision (DS) across all four stages of the decoding process, as shown in Figure 2. Figure 6 shows the detailed construction. Our deep supervision block comprises a residual block, two 1×1 convolution layers, and an upsampling layer with bilinear operation for enlarging the feature map. Deep supervision effectively directs the learning of features in the intermediate layers, guided directly by loss functions and corresponding labels. We perform upsampling on features from the initial four hidden stages, aligning them with the dimensions of the final prediction stage. Subsequently, we use the Dice loss functions to supervise these stages. After decoding, the final output is rescaled to match the original input dimensions. This rescaled output is then processed through a softmax layer to generate the distribution of class probabilities. It is important to note that deep supervision is not employed during the inference stage. In this phase, only the last layer of the decoder is utilized to generate the segmentation prediction.

### 2.7. Loss Function

The Dice loss serves as a conventional loss function in image segmentation tasks, quantifying the disparity between the predicted mask and the ground-truth mask, as established in [30]. However, certain limitations persist when employing this function. Notably, in the absence of a segmentation target, the Dice loss yields a score of 0. This signifies that the Dice loss function does not penalize false positives.

To address this issue, we employ the enhanced class-wise Dice loss function to compute Dice Similarity Coefficients (DSCs) for background and SRC segmentation in benign and malignant images, respectively, as detailed in [31]. This refined loss function effectively mitigates false positives, underscoring its practical utility in clinical applications. The enhanced class-wise dice loss (CDL) function is detailed by:

(2) $$L_{\mathrm{CDL}}=1-\sum_{i}^{N}\left(y_{p}\frac{y_{i}\hat{y_{i}}}{y_{i}+\hat{y_{i}}}+\frac{\left(1-y_{p}\right)\left(1-y_{i}\right)\left(1-\hat{y_{i}}\right)+\epsilon }{\left(1-y_{i}\right)+\left(1-\hat{y_{i}}\right)+\epsilon }\right),$$

where $y_{i}$ represents the binary label for pixel *i*, $\hat{y_{i}}$ corresponds to the predicted probability, and *N* denotes the total pixel count within a patch. The parameter ϵ is introduced as a small value to prevent division by zero.

The assignment of a patch label ($y_{p}$) hinges on the presence or absence of a lesion area. The employment of the $L_{\mathrm{CDL}}$ loss function effectively mitigates pixel-level class imbalance, leading to the generation of an all-zero mask during training for negative samples.

## 3. Experiments

This section describes our experiments designed to assess and appraise the segmentation performance of the proposed approach. In particular, we provide an elaborate account of our SRC dataset, evaluation metrics, and implementation specifics.

### 3.1. Dataset

In our experiments, we employed the SRC dataset to train and validate our model sourced from two organs: the gastric mucosa and intestine. Our dataset was comprised of 308 high-resolution images, with 77 positive and 231 negative samples. These positive samples were cropped from 20 whole slide images (WSIs), all of which are comprehensively annotated. Each WSI was stained with H&E, scanned at a ×40 magnification and sourced from two organs: the gastric mucosa and intestine. Experienced pathologists identified and labeled each signet ring cell using the labelme, ensuring accuracy with a precise ground truth surrounding each cell. For our proposed model, we selected 62 positive and 186 negative samples from our dataset for training. During the training process, we also used 7 positive and 21 negative samples for validation. To assess the effectiveness of our model, we employed 8 negative and 24 positive samples as test data.

To demonstrate our proposed method’s generalizability and its performance in different contexts, we used the GlaS dataset to verify the network. Glands represent pivotal histological structures found across various organ systems, primarily responsible for the secretion of proteins and carbohydrates. Adenocarcinomas, malignant tumors originating from glandular epithelium, stand out as the most prevalent form of cancer. Pathologists routinely rely on gland morphology to assess the malignancy levels of various adenocarcinomas in organs such as the prostate, breast, lung, and colon. Accurate gland segmentation is imperative for acquiring dependable morphological data. However, this task can be challenging due to the diverse glandular morphologies present across different histological grades. The GlaS dataset comprises a total of 165 tissue sections, encompassing both positive and negative samples. Within this dataset, our training subset contained 85 samples, with an additional 17 samples reserved for the validation set. Furthermore, the GlaS dataset offers two distinct test sets, denoted as testA and testB, consisting of 60 and 20 samples, respectively. We employed the validation set to identify the optimal model, conducting all performance evaluations on the combined results from testA and testB.

**Figure 7:**
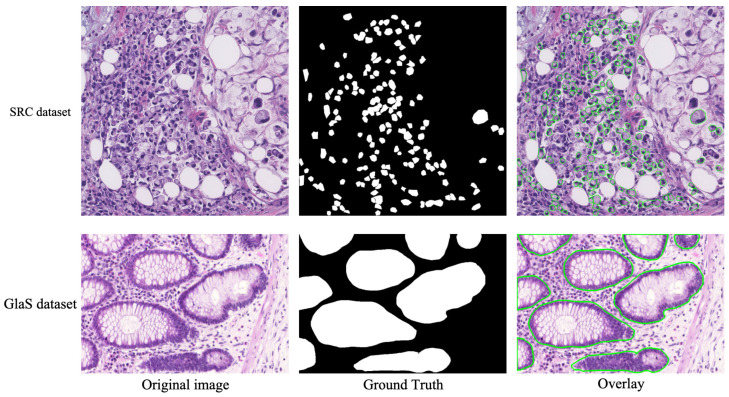
Two samples from the SRC and GlaS datasets.

Two examples from the SRC and GlaS datasets are illustrated in Figure 7. Notably, most previous studies have concentrated on gland segmentation within either healthy or benign samples, often overlooking intermediate or high-grade cancers. Consequently, these studies frequently tailor their methods to specific datasets.

### 3.2. Evaluation Metrics

In the context of evaluating segmented models, pixel-based metrics are often employed for assessing accuracy. We use a variety of metrics to evaluate the performance of our network, including the Dice similarity coefficient (DSC), Jaccard index, precision, and recall.

While both DSC and Jaccard are used to measure the similarity between predicted and labeled images, they have distinct focuses. Jaccard measures the consistency of extracted features and is suitable for comparing similarities and differences between limited sample sets. In contrast, DSC is more sensitive to the inner padding of the mask and is primarily used to calculate the similarity of two sets, making it our primary performance indicator.

In addition to DSC and Jaccard, we also employ precision and recall to evaluate our network’s performance. Precision measures the proportion of predicted targets that are accurately identified, while recall represents the number of actual targets correctly identified based on predicted results.

Overall, these metrics allow us to comprehensively assess the accuracy of our segmentation network in identifying and classifying targets in the SRC dataset. These metrics are formulated as follows:

(3) $$DSC=\frac{2TP}{FP+2TP+FN},$$

(4) $$Jaccard=\frac{TP}{FP+TP+FN},$$

(5) $$Precision=\frac{TP}{FP+TP},$$

(6) $$Recall=\frac{TP}{TP+FN},$$

where *TP*, *FP*, and *FN* correspond to the true positive predictions, false positive predictions, and false negative predictions, respectively.

### 3.3. Implementation Details

Our proposed method was implemented using PyTorch 1.8.0 and trained on a single NVIDIA GeForce RTX 3090 GPU. The initial learning rate was set to $1.0\times 10^{-4}$. We employed the Adam optimizer for training the algorithm on the SRC dataset, with momentum and weight decay values of 0.99 and $1\times 10^{-8}$, respectively.

For our SRC dataset, input images were densely cropped into patches with 128×128 pixels. The training process consisted of 2000 epochs with a batch size of 4. Data augmentation techniques included Gaussian blur, hue and saturation adjustments, affine transformations, as well as horizontal and vertical flips.

## 4. Discussion and Analysis

### 4.1. Discussion on Different Blocks

Table 1 presents the outcomes of an ablation study, illustrating the improvements in performance resulting from the integration of various blocks into the UNet architecture. These integrated blocks include ResGhost, GCA, and DS. It is evident that ResGhost, GCA, and DS all contribute to the enhancement of model performance. Our proposed RGGC-UNet, in particular, achieves the highest DSC. Furthermore, we conduct a detailed analysis of the performance of different discriminators in the context of the RGGC-UNet architecture. The corresponding results are provided in Table 2.

### 4.2. Comparison on SRC Dataset

Table 2 provides a comparative analysis of the performance between our proposed model and other popular models, using four metrics on our SRC dataset. The results clearly indicate that our proposed model achieves the highest scores in terms of DSC, Jaccard, recall, and precision. In all four metrics, our model outperforms the alternatives significantly.

Table 3 presents an overview of the computational complexity in terms of FLOPS and parameters. Although our proposed model may not boast the minimum number of FLOPS or parameters compared to other popular models, it effectively strikes a balance between computational load and model size. Consequently, our network represents an advantageous trade-off between accuracy and efficiency.

**Figure 8:**
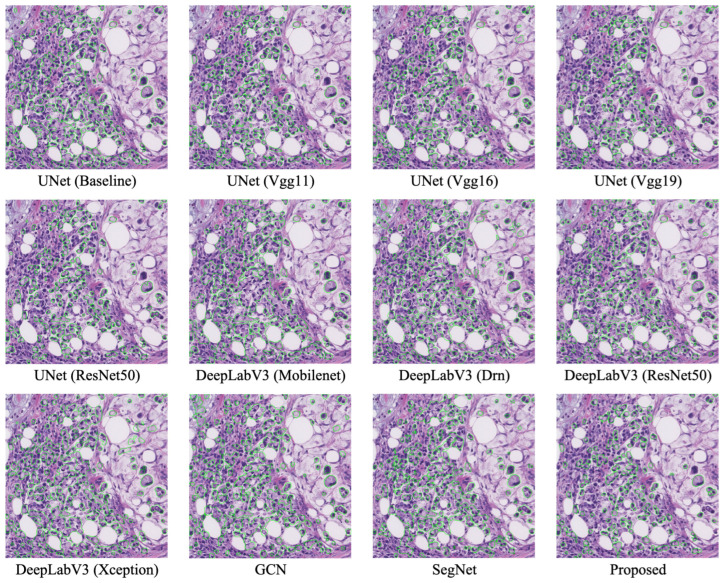
Segmentation results of various models on the SRC dataset.

Figure 8 visually displays the segmentation results of various models, including ours and the findings of [12,19,31,32,33,34,35,36] on the SRC dataset. The visual evidence demonstrates that our model provides the most optimal alignment between its predictions and the ground truth. In comparison to other leading networks, our model excels in successfully segmenting SRCs. Overall, our proposed model excels at distinguishing between clustered and overlapping cells, achieving state-of-the-art accuracy in SRC segmentation tasks.

### 4.3. Comparison on GlaS Dataset

**Figure 9:**
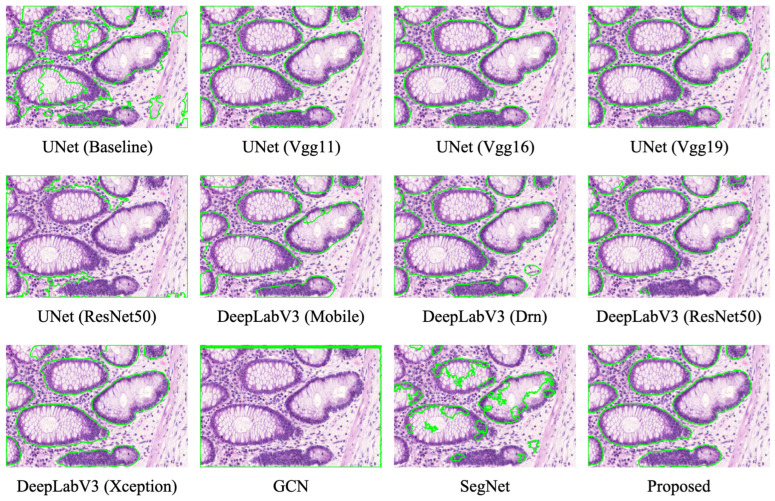
Segmentation results of different models on the GlaS dataset.

To illustrate the generalizability of our proposed method and its performance under different scenarios, we also validate the network using the GlaS dataset. As demonstrated in Table 4, our proposed network consistently outperforms other methods in gland segmentation tasks, achieving the highest scores. Figure 9 visually presents the results of gland segmentation using various models on the test set. The visual evidence underscores that our proposed network effectively segments gland boundaries and attains superior DSC, Jaccard, precision, and recall. Our innovative approach has direct applicability in computer-aided pathological diagnosis systems, potentially alleviating the workload of pathologists.

## 5. Conclusions

In this research, we have developed RGGC-UNet, an efficient and accurate deep learning framework specifically designed for the semantic segmentation of SRCs in pathological images. The central component of our model lies in its encoder-decoder architecture, where we have introduced an innovative encoder. This encoder is purposefully crafted to adeptly capture features, preserving the relationships between distant pixels. Particularly noteworthy is our introduction of the ghost coordinate attention mechanism, which inherits the advantages of coordinated attention. It adeptly models inter-channel relationships while simultaneously capturing long-range dependencies with precise positional information and ghost blocks.

To assess the effectiveness of RGGC-UNet, we conducted extensive experiments on a dataset that we curated. The results indicate that our proposed model can surpass leading models in terms of segmentation accuracy and efficiency, benefiting from ghost block and ghost coordinate attention. An important attribute of our proposed framework is its adaptability; it can seamlessly transition to other tasks related to pathological image analysis. Furthermore, the decoder structure we have presented exhibits flexibility and can be integrated into other deep convolutional neural networks dedicated to pathological image analysis.

Nonetheless, it is important to acknowledge certain limitations. We have yet to evaluate the performance of our model on natural images, leaving its effect in such contexts uncertain. Recognizing this as an existing challenge, our future research endeavors will involve an in-depth theoretical analysis to provide more robust insights.

## Figures and Tables

**Table 1:** Performance gain by integrating different blocks into UNet on the SRC dataset. The best results are indicated in bold.

UNet	ResGhost	GCA	DS	DSC
√				0.5298
√			√	0.5621
√	√			0.5635
√	√		√	0.5827
√	√	√		0.7231
√	√	√	√	**0.7852**

**Table 2:** Comparative results for signet ring cell segmentation on the proposed dataset. The best results are indicated in bold.

Method	DSC	Jaccard	Precision	Recall
UNet(Baseline) [19]	0.5621	0.4007	0.5160	0.6434
UNet(Backbone: Vgg11) [32]	0.5771	0.4160	0.5530	0.6271
UNet(Backbone: Vgg16) [33]	0.5817	0.4191	0.5599	0.6304
UNet(Backbone: Vgg19) [31]	0.5850	0.4232	0.5930	0.6036
UNet(Backbone: ResNet50) [34]	0.5531	0.3943	0.6512	0.5316
DeepLabV3(Backbone:Mobilenet) [35]	0.4620	0.3098	0.3320	0.7804
DeepLabV3(Backbone: Drn) [35]	0.4564	0.3035	0.3361	0.7340
DeepLabV3(Backbone: ResNet50) [35]	0.5200	0.3576	0.4210	0.6916
DeepLabV3(Backbone: Xception) [35]	0.5227	0.3599	0.4020	0.7572
GCN [36]	0.4574	0.3026	0.3691	0.6270
SegNet [12]	0.4728	0.3198	0.4084	0.5867
Proposed	**0.7852**	**0.6482**	**0.7800**	**0.7964**

**Table 3:** Number of the FLOPS and parameters.

Model	GFLOPS	Params (M)
UNet (Baseline) [19]	16.70	14.50
UNet (Backbone: Vgg11) [32]	17.66	17.47
UNet (Backbone: Vgg16) [33]	22.79	22.96
UNet (Backbone: Vgg19) [31]	25.51	28.27
UNet (Backbone: ResNet50) [34]	55.87	59.04
DeepLabV3 (Backbone: Mobilenet) [35]	4.45	7.55
DeepLabV3 (Backbone: Drn) [35]	23.31	40.73
DeepLabV3 (Backbone: ResNet50) [35]	11.06	59.22
DeepLabV3 (Backbone: Xception) [35]	10.33	54.5
GCN [36]	7.64	58.25
SegNet [12]	20.06	29.44
Proposed	51.86	48.03

**Table 4:** Comparative results for gland segmentation on the Glas dataset. The best results are indicated in bold.

Method	DSC	Jaccard	Precision	Recall
UNet(Baseline) [19]	0.5132	0.3745	0.9285	0.3549
UNet(Backbone: Vgg11) [32]	0.7486	0.6195	0.9313	0.6268
UNet(Backbone: Vgg16) [33]	0.7324	0.6038	0.8375	0.6507
UNet(Backbone: Vgg19) [31]	0.7289	0.600	0.7928	0.6747
UNet(Backbone: ResNet50) [34]	0.6511	0.5065	0.9375	0.4985
DeepLabV3(Backbone:Mobilenet) [35]	0.6839	0.5410	0.9367	0.5388
DeepLabV3(Backbone: Drn) [35]	0.7367	0.6039	0.9375	0.6065
DeepLabV3(Backbone: ResNet50) [35]	0.6887	0.5503	0.9358	0.5203
DeepLabV3(Backbone: Xception) [35]	0.6867	0.5564	0.9342	0.5430
GCN [36]	0.5696	0.4220	0.7863	0.4464
SegNet [12]	0.5206	0.3799	0.9445	0.3592
Proposed	**0.9571**	**0.9190**	**0.9548**	**0.9611**

## Data Availability

The data that support the findings of this study are available from the corresponding author upon reasonable request.
